# Headaches*Translated by Dr. S. Lilienthal, from the German of Dr. Kunkel, Kiel.

**Published:** 1885-06

**Authors:** 


### CLINICAL BUREAU.

### HEADACHES.*

A widow, over sixty, suffered for three months from frontal headache on the left side of the occiput, pain in os zygomaticum here and there, changing rapidly its seat. The pain is the same by day and by night, setting in suddenly, *shooting in.* Sometimes nausea, but not during the paroxysms. *Cannot lie down with the head low;* when patient looks downward, yellow stars; looking at something white, *red* flowers before the eyes. June 15th, 1875, Spigelia$^{200}$ (Lehrman), one dose.

The patient was for a whole year free from pain. September 23d, 1876, she complains of headache in forehead, temples, teeth, *deep in the orbits,* and about midnight. Cannot lie down with her head low. Another dose of Spigelia$^{200}$.

November 22d, 1878.—For the last ten days the old pains; in windy weather, but can lie low; bitter taste. Spigelia$^{30}$, three powders, every evening a powder. So far she has not yet returned.

In *Spigelia* the pain is mostly lancinating, tearing, attacks suddenly, and more frequently the left than the right side of the face, teeth, zygoma (Staph., Stram., Sep.), the bulbs, and the orbits. It appears mostly at irregular intervals. Sometimes the painful parts are swollen, *in windy weather, when lying with the head low, when lying on the left side, on stooping.* Simultaneously we may meet palpitations with or without the pains; pupils mostly dilated. Spigelia is especially efficacious in so-called pure neuralgia, and the patients often think that there is nothing the matter with them except those pains.

*Arsenicum* removes sometimes semilateral headaches. Here, also, agg. by *wind, before and during east winds, and when lying with the head low, regular periodicity of the attacks, and after midnight.* Pain, especially burning or beating. Complications: sleeplessness; anguish, especially when alone; palpitations; thirst, with frequent sipping; tenesmus urinæ—slow, scanty discharge, with burning in the urethra.

*Platina* has headache in the evening, after lying down; *before or during windy weather, and when lying on left side; in fresh air,* and therefore desire for it. The pain is squeezing, stitching, boring, drawing, with sensation of numbness. The nervous

* Translated by Dr. S. Lilienthal, from the German of Dr. Kunkel, Kiel.

pains are often caused by emotions, fright, anger. Impulses of the mind constantly changing without cause, jumping from one extreme to another; spasmodic gaping without sleepiness.

*Phosphorus* has, like Spigelia, the suddenly appearing (shooting in) pains; with Platina and Spigelia, the aggravation *when lying on left side, in windy weather or before it.* Sleepiness in daytime, night-sweats during sleep, which pass off as soon as he awakes; anguish, with or without increased sleepiness; during thunderstorms, vertigo, veil before eyes.

*Phosphoricum acidum* has also sleepiness in daytime and great prostration, like Phosphorus; from emotions; tendency to painless diarrhœa, with green or gray stools; disgusting pappy taste; urine watery or murky and of a foul odor. The excellent effect of Phosphoric acid in some protracted gonorrhœa with great prostration in cases of vesical catarrhs is well known. The drug stands in close correlation to Thuja.

*Calcarea* is a capital remedy in left-sided headaches, semilateral specially. Obese people, with blonde hair and tendency to perspire, are more affected. Wide pupils; glandular swellings after catching cold, cardialgia, with bloated pit of stomach and where the pressure of the clothing cannot be borne; profuse anteponing menses, preceded by leucorrhœa, agg. by drafts and dampness.

*Kali carbonicum.*—The pain is tearing, stitching, either on one or the other side. It acts capitally in sequelæ of scarlatina or measles, or after puerperium, agg. by draft and coldness; attacks at night, mostly after midnight, about 2 or 3 A.M., often at the same time. Constipation, with large-formed stools, hemorrhoids, renal affections, phthisical disposition, tendency to œdema of the face. Hemicrania is justly often considered a complication of renal affections. (Kali, Coloc., Phos. ac.)

*Colocynth* acts well where the headache is caused by anger, depressing emotions, but *improvement by lying on affected side.*

In scorbutic Staphisagria we also meet the mental depression. Nothing new, but it is personal experience, and for that purpose given.

---

I. H. A. NOTICE.—Any physicians wishing to become members of the International Hahnemannian Association will please send their applications to the Chairman of the Board of Censors by or before the next Annual Meeting of the Association, on the 23d of June, at Syracuse, N. Y. Blank applications for membership may be had by addressing the Secretary, Dr. J. B. G. Custis, 604 East Capitol Street, Washington, D. C., or of the Chairman of the Board of Censors. The application must be indorsed by three members of the Association and accompanied by two dollars, for initiation fee and dues for one year. C. Pearson, M. D., Chairman Board of Censors, 611 Twelfth Street, Washington, D. C.

